# Internet Addiction and Gaming Disorder During the COVID-19 Pandemic Among Young People in Southern Karnataka

**DOI:** 10.7759/cureus.42159

**Published:** 2023-07-19

**Authors:** Narendra Babu Mokshathaa, S Vishwas

**Affiliations:** 1 Community Medicine, Sri Devaraj Urs Academy of Higher Education and Research, Kolar, IND; 2 Epidemiology and Public Health, Sri Devaraj Urs Academy of Higher Education and Research, Kolar, IND

**Keywords:** young people, mental health, covid-19, internet gaming disorder, internet addiction

## Abstract

Background: Advances in technology and increased accessibility to modern gadgets have opened up new options for giving alternate recreational activities. The country has seen rapid growth in the smartphone market over the past decade, and mobile phones are preferred by the vast majority of gamers over traditional personal computers and laptops. The coronavirus disease 2019 (COVID-19) pandemic and related restrictions, online teaching, and the use of the internet for work have forced people to resort to the internet more than during the pre-pandemic period. There have not been many studies done to evaluate internet addiction (IA) and internet gaming disorder (IGD) among young people in India, and research is needed to quantify the magnitude of the problem and undertake timely public health actions.

Methods: A cross-sectional study was conducted among 400 young people aged 10 to 24 years in Kolar district. Young people meeting the eligibility criteria from schools and colleges were randomly selected to include 67 participants per taluk. Standardized validated nine-item Internet Gaming Disorder Scale-Short-Formand 20-item Internet Addiction Test questionnaire were used to collect data. Data were entered using EpiData version 3.1 (EpiData Association, Odense, Denmark) and analyzed using IBM SPSS version 20 (IBM Corp., Armonk, NY). A p-value < 0.05 was considered statistically significant.

Results and conclusion: COVID-19 control measures have caused more young people to access the internet in the recent past. Among young people studied, 58.5% and 6.5% have IA and IGD, respectively. Factors like living in urban areas, belonging to families above the poverty line, not living with parents, years of internet use, and increased access to internet/gadgets during the COVID-19 pandemic were significantly associated with IA and IGD. Since addiction to the internet and online gaming is known to have a negative impact on the mental health and well-being of young people, in light of IGD being listed in the International Classification of Diseases, there is an urgent need to target these conditions, especially for young people.

## Introduction

With progressive adaptation to advances in technology, internet use has become an integral part of human life. The availability of modern gadgets and increased access to the internet have made humans more dependent on the internet than ever. In India, those between the age group of 12 and 34 years dominate internet use, which accounts for 65% of the total internet usage [1]. Accessibility to the internet has drastically improved, such that there has been an increased dependency on the internet for information by students.

Ever-evolving digital lifestyle has opened up new options for giving alternate recreational activities to people who do not want to play physical games. As a result, online gaming has become an engaging and viable platform for entertainment and professional gaming. Broad internet coverage, combined with the growing use of cell phones, laptops, tablets, and specialized gaming consoles, has fuelled an era of online gaming that simulates near-real-life gameplay and provides a deeply engaging experience for the user. The country has seen rapid growth in the smartphone market over the past decade, and mobile phones are preferred by the vast majority of gamers [1]. Around 55% of casual gamers and 66% of hard-core gamers in India were under the age of 24 years. According to a report by the All India Gaming Federation, the number of young gamers is expected to grow further [2].

The coronavirus disease 2019 (COVID-19) pandemic has tremendously changed the way humans interact with each other. The lockdowns and social distancing norms had forced people to resort to the internet more than during the pre-pandemic period. Schools and colleges have started online teaching and those who otherwise were less likely to use the internet for work have found it more comfortable to work with the internet. Also, because of government regulations restricting the movement of people in public places, young people have resorted to online gaming and social media for entertainment and social connection. As a result, there is a drastic rise in online gaming among young people [3].

Internet gaming disorder (IGD) has been recognized as a clinical disorder and is listed in the 11th revision of the International Classification of Diseases [4] by the World Health Organization and in the fifth edition of the Diagnostic and Statistical Manual of Mental Disorders by the American Psychiatric Association [5,6]. Gaming disorder is defined as a pattern of gaming behavior characterized by impaired control over gaming, increasing priority given to gaming over other activities to the extent that gaming takes precedence over other interests and daily activities, and continuation or escalation of gaming despite the occurrence of negative consequences [7]. IGD has been shown to have associations with aggression, neuroticism, introversion, avoidant and schizoid interpersonal tendencies, boredom inclination, social inhibition, narcissistic personality traits, anxiety, lower self-esteem, and emotional intelligence. Like other mental health illnesses, the identification and management of individuals with IGD in the community is a challenge. Factors such as the lack of data on the exact magnitude of the problem, the ubiquity of the internet, access to smartphones from a young age, peer pressure, virtual rewards inbuilt into the game, multiplayer online tournaments, and competitions add to the complexity of the problem [8,9].

Addiction to internet games is harder to treat as compared to addiction to alcohol or drugs [10]. The disorder has compelled some of the players to take extreme steps, and there are instances of such internet games claiming the lives of young gamers in India. The Karnataka government banned gambling, including games of skill, in 2021. Although some studies done in different states in India reported the prevalence of internet addiction (IA) to be between 20% and 40%, these rates were during the pre-pandemic period [11], and there are limited studies during the COVID-19 pandemic and additional research is urgently needed in this field to quantify the magnitude of the problem and undertake relevant public health actions. Hence, this study was undertaken to study the prevalence and associated factors of IA and IGD among young people in Kolar.

## Materials and methods

A cross-sectional study was carried out over a period of six months (July 2022 to January 2023) among young people aged 10 to 24 years in the Kolar district. Although during this period of our study, the number of cases of COVID-19 in the country had reduced, COVID-19 continued to be a public health emergency of international concern [12], and our study questionnaire focused mainly on the students' experience during the COVID-19 lockdowns.

The sample size estimated considering the prevalence proportion to be 50% with an absolute error of 5% was 400. A list of schools and colleges in the district where young people aged 10 to 24 years are enrolled was obtained from the District Education Department. From this list, schools and colleges were selected by simple random sampling technique till a sample of 67 eligible participants was included from each of the six blocks in the district. All the eligible participants present in the school/college at the time of the interviewer's visit were included in the study. Any student who does not have access to a smartphone, persons with visual or auditory impairment, and/or diagnosed with any neuropsychiatric disorder were excluded from the study.

A validated structured questionnaire, which was translated into the local language, was administered directly by a trained interviewer. The questionnaire included information on the basic demographic factors of the participants and key factors associated with IA and gaming. Standardized validated nine-item Internet Gaming Disorder Scale-Short-Form (IGDS9-SF) [13] and 20-item Internet Addiction Test (IAT) questionnaire [14] were used to collect information. Students identified with IGD were referred to a psychiatrist and given necessary counseling.

Informed written consent was obtained from study participants and their parents. Formal approval from school/college authorities was also obtained prior hand. Data collected were double-checked for errors and were entered and validated using EpiData version 3.1 (EpiData Association, Odense, Denmark). Data were analyzed using IBM SPSS version 20 (IBM Corp., Armonk, NY). Continuous data were expressed using mean (SD). All categorical data were expressed in counts (percentage). The association between variables was tested using the chi-square test. A p-value < 0.05 was considered statistically significant.

## Results

About half of the study participants were in the age group of 15 to 19 years and many of them (62.5%) were residing in urban areas. While there was not much of a difference in the proportion of those belonging to different social classes, the majority of study participants were Hindus and belonged to nuclear families. Among the study participants, 58 had been diagnosed with COVID-19 in the past. The majority of students (86.8%) reported that their school/college had adopted online teaching during the pandemic and 78.5% reported that they have increased access to the internet and gadgets since the COVID-19 pandemic (Table 1).

**Table 1:** Distribution of study participants based on COVID-19 status and internet use (N = 400)

Characteristics		Number	Percentage
Ever been diagnosed with COVID-19?	Yes	58	14.5
	No	342	85.5
School/college adopted online teaching during the COVID-19 pandemic?	Yes	347	86.8
	No	53	13.2
Increased access to the internet/gadgets during the COVID-19 pandemic?	Yes	314	78.5
	No	86	21.5
Years of internet use	0 to 3 years	237	59.3
	4 to 7 years	121	30.3
	8 to 11 years	32	8.0
	12 to 15 years	9	2.3
	16 years and more	1	0.3
Use of the internet for academic purposes (n, %)		358 (89.5)	

**Figure 1:**
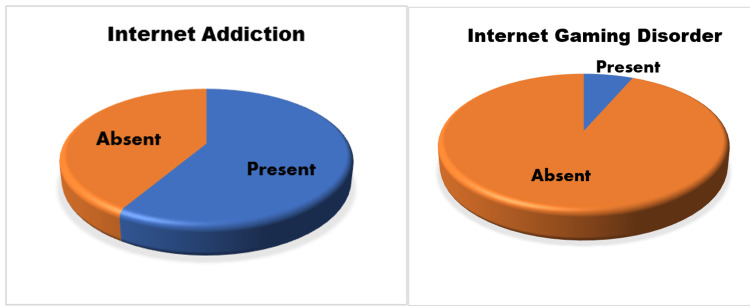
Prevalence of internet addiction and internet gaming disorder among study participants (N = 400)

**Figure 2:**
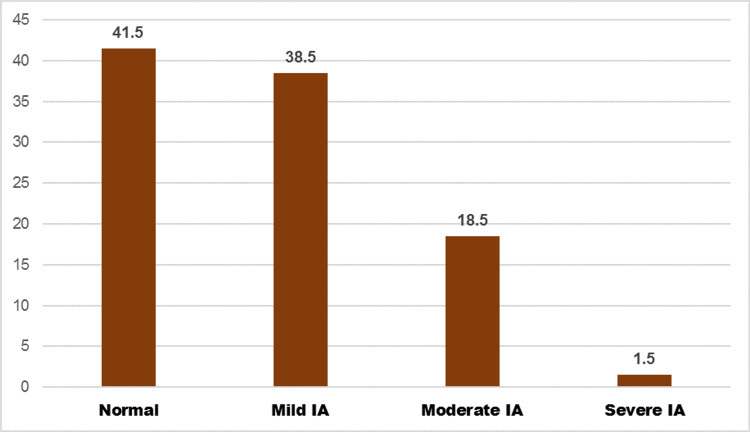
Distribution of study participants by severity of internet addiction (percentage) Internet Addiction Test scores of 0 to 30 are considered normal, 31 to 49 are considered mild, 50 to 79 are considered moderate, and 80 to 100 are considered severe [14]. IA: internet addiction.

The mean number of years of internet use among study participants was 3.97 years (SD = 2.79) and most of them (89.6%) were using the internet for seven years. The majority of students (89.5%) were using the internet for academic purposes as well. Our study identified that 234 study subjects (58.5%) had IA and 26 of them (6.5%) had IGD since their IAT scores and nine-item IGDS9-SF scale scores were more than the cut-off values (Figures 1, 2).

The prevalence of IA in relation to various factors was studied (Table 2) and a significant difference in IA was noted among different age groups, urban students, students belonging to families below the poverty line, students residing with parents, those with a lower number of years of internet use, and among those who had increased access to gadgets and internet during the COVID-19 pandemic. Of the study subjects, 41.5% had IAT scores within normal limits. Of the study subjects, 38.5%, 18.5%, and 1.5% had mild, moderate, and severe IA, respectively. Similarly, for IGD (Table 3), the prevalence was significantly different for those with different years of internet use and among those with increased access to the internet and gadgets during the COVID-19 pandemic.

**Table 2:** Prevalence of internet addiction among study participants in relation to various factors (N = 400)

Variable	Internet addiction		Total, n (%)	Chi-square (p-value)
	Present, n (%)	Absent, n (%)		
Age category (years)				9.934 (<0.05)
10 to 14	74 (63.8)	42 (36.2)	116 (29)	
15 to 19	93 (50.3)	92 (49.7)	185 (46.3)	
20 to 24	67 (67.7)	32 (32.3)	99 (24.7)	
Gender				0.415 (0.5)
Male	132 (57.1)	99 (42.9)	231 (57.8)	
Female	102 (60.4)	67 (39.6)	169 (42.2)	
Locality				7.142 (<0.05)
Rural	75 (50)	75 (50)	150 (37.5)	
Urban	159 (63.6)	91 (36.4)	250 (62.5)	
Social class				6.216 (<0.05)
Above poverty line	122 (64.6)	67 (35.4)	189 (47.7)	
Below poverty line	108 (52.2)	99 (47.8)	207 (52.3)	
Residing with parents				4.802 (<0.05)
Yes	134 (54.3)	113 (45.7)	247 (61.8)	
No	100 (65.4)	53 (34.6)	153 (38.2)	
Mother’s education				14.959 (0.05)
Illiterate/primary/middle school	15 (32.6)	31 (67.4)	46 (11.5)	
High school	65 (59.1)	45 (40.9)	110 (27.5)	
Intermediate/diploma	45 (61.6)	28 (38.4)	73 (18.2)	
Graduate	66 (63.5)	38 (36.5)	104 (26.0)	
Masters and above	43 (64.2)	24 (35.8)	67 (16.8)	
Years of internet use				18.096 (<0.001)
0 to 3 years	120 (50.6)	117 (49.4)	237 (59.25)	
4 to 7 years	80 (66.1)	41 (33.9)	121 (30.25)	
8 to 11 years	25 (78.1)	7 (21.9)	32 (8)	
12 years and above	9 (90)	1 (10)	10 (2.5)	
Ever been diagnosed with COVID-19?				1.376 (0.24)
Yes	38 (65.5)	20 (34.5)	58 (14.5)	
No	196 (57.3)	146 (42.7)	342 (85.5)	
Online classes during the COVID-19 pandemic				1.437 (0.23)
Yes	207 (59.7)	140 (40.3)	347 (86.8)	
No	27 (50.9)	26 (49.1)	53 (13.2)	
Increased access to the internet/gadgets during the COVID-19 pandemic				14.301 (<0.001)
Yes	199 (63.4)	115 (36.6)	314 (78.5)	
No	35 (40.7)	51 (59.3)	86 (21.5)	

**Table 3:** Prevalence of internet gaming disorder among study participants in relation to various factors (N = 400)

Variable	Internet gaming disorder		Total, n (%)	Chi-square (p-value)
	Present, n (%)	Absent, n (%)		
Age category (years)				0.427 (0.8)
10 to 14	9 (7.8)	107 (92.2)	116 (29)	
15 to 19	11 (5.9)	174 (94.1)	185 (46.3)	
20 to 24	6 (6.1)	93 (93.9)	99 (24.7)	
Gender				0.164 (0.7)
Male	16 (6.9)	215 (93.1)	231 (57.8)	
Female	10 (5.9)	159 (94.1)	169 (42.2)	
Locality				0.099 (0.75)
Rural	9 (6)	141 (94)	150 (37.5)	
Urban	17 (6.8)	233 (93.2)	250 (62.5)	
Social class				0.058 (0.81)
Above poverty line	13 (6.9)	176 (93.1)	189 (47.7)	
Below poverty line	13 (6.3)	194 (93.7)	207 (52.3)	
Residing with parents				0.194 (0.66)
Yes	15 (6.1)	232 (93.9)	247 (61.8)	
No	11 (7.2)	142 (92.8)	153 (38.2)	
Mother’s education				1.605 (0.8)
Illiterate/primary/middle school	4 (8.7)	42 (91.3)	46 (11.5)	
High school	5 (4.5)	105 (95.5)	110 (27.5)	
Intermediate/diploma	6 (8.2)	67 (91.8)	73 (18.2)	
Graduate	6 (5.8)	98 (94.2)	104 (26.0)	
Masters and above	5 (7.5)	62 (92.5)	67 (16.8)	
Years of internet use				10.184 (0.017)
0 to 3 years	16 (6.8)	221 (93.2)	237 (59.25)	
4 to 7 years	6 (5)	115 (95)	121 (30.25)	
8 to 11 years	1 (3.1)	31 (96.9)	32 (8)	
12 years and above	3 (30)	7 (70)	10 (2.5)	
Ever been diagnosed with COVID-19?				1.650 (0.19)
Yes	6 (10.3)	52 (89.7)	58 (14.5)	
No	20 (5.8)	322 (94.2)	342 (85.5)	
Online classes during the COVID-19 pandemic				2.336 (0.12)
Yes	20 (5.8)	327 (94.2)	347 (86.8)	
No	6 (11.3)	47 (88.7)	53 (13.2)	
Increased access to the internet/gadgets during the COVID-19 pandemic				5.135 (0.02)
Yes	25 (8)	289 (92)	314 (78.5)	
No	1 (1.2)	85 (98.8)	86 (21.5)	

## Discussion

After the first case of COVID-19 reported in India in January 2020, the country has seen three major waves of COVID-19 to date with a peak number of cases during September 2020, May 2021, and January 2022. There were lockdowns, restrictions of movement, and social distancing norms imposed to tackle the spread of disease. As a consequence of these measures, there is a tremendous change in the way the country’s young population uses technology and the internet.

In this study, we discovered that 58.5% of participants exhibited an addiction to the internet. Based on the cut-off scores of 50 and 40 on Young's IAT score, a systematic analysis of 50 research studies conducted in 19 states of India between 2010 and 2020 estimated the total prevalence of IA to be 19.9% and 40.7%, respectively. Young's IAT scores between 0 and 30 are considered to reflect normal levels of internet usage, scores between 31 and 49 reflect mild, 50 to 79 reflect moderate, and 80 to 100 reflect severe levels of dependence. The prevalence of IA in our study even with the mildest addiction is more than that reported in the systematic review. This could be attributed to a significant increase in internet use during the pandemic period.

In comparison to studies conducted among students in other parts of the world, our study found a higher prevalence of IA. In our study, the prevalence of IA was found to be 63.8%, 50.3%, and 67.7%, respectively, in the age categories of 10 to 14, 15 to 19, and 20 to 24 years. The vast majority of India’s population is distributed in productive years with a median age of 20 years and it is fairly low compared to other countries of the world. This age distribution might be a factor contributing to the increasing number of young people being addicted to the internet and in turn, contributing to a higher prevalence of IA in India compared to other countries [15-18].

In this study, IGD was shown to be 6.5%. A systematic evaluation of 5550 pertinent papers reported an incidence of IGD ranging from 0.21% to 57.50%. According to a cross-sectional study conducted on 142 students in Maharashtra, 10.6% of 13 to 19-year-old pupils had IGD. This research was conducted among students in grades 8-12 [19]. Our study has found a lower prevalence of IGD probably because of the broader age group included and predominantly rural students in this part of Kolar. But our study has found that with increasing access to the internet and the growing number of years of internet use, IGD significantly increased.

Limitations of the study

Although possible sources of bias have been eliminated in our study in which the questionnaire was translated into a simple understandable local language and was administered individually by the interviewer, the possibility of peer influence cannot be ruled out as data acquisition was done at school/college.

## Conclusions

IGD is identified as a major public health problem among adolescents and youth. COVID-19 control measures have caused more and more young people to access the internet in the recent past. Besides being a mental health threat for young people, individuals with IGD are generally unable to limit the time they spend gaming and continue to play despite negative consequences, including impulsive illegal actions, and it also affects the social behavior and scholastic performance of young people. The findings of this study reveal that more than half of the study population and about 7% of them have IA and IGD, respectively. Although other modes of gaming apart from that of mobile phone gaming were not reported and the problem of IGD was relatively lower in this study, there is a need to address the growing problem of IA among young people.
